# Predictors of failure of intersegmental line creation using bronchoscopic jet ventilation for thoracoscopic pulmonary segmentectomy

**DOI:** 10.1186/s40981-021-00457-5

**Published:** 2021-07-06

**Authors:** Mizuko Ikeda, Miwako Tanabe, Ayumi Fujimoto, Tomoka Matsuoka, Makoto Sumie, Ken Yamaura

**Affiliations:** 1 Department of Anesthesiology, Federation of National Public Service Personnel Mutual Aid Associations, Fukuoka, Japan; 2 Present Address: Section of Anesthesiology, Department of Diagnostics and General Care, Fukuoka Dental College, 2-15-1, Tamura, Sawara-ku, Fukuoka, Fukuoka 814-0193 Japan; 3 Present Address: Department of Anesthesiology, St. Mary’s Hospital, Fukuoka, Japan; 4 Department of Anesthesiology and Critical Care Medicine, Kyushu University Hospital, Fukuoka, Japan; 5 Department of Anesthesiology and Critical Care Medicine, Graduate School of Medical Science, Kyushu University, Fukuoka, Japan

**Keywords:** Thoracoscopic surgery, Pulmonary segmentectomy, Broncoscopy, Jet ventilation

## Abstract

**Background:**

During pulmonary segmentectomy, identification of the target segment is essential. We used bronchoscopic jet ventilation (BJV) to delineate the intersegmental plane by selectively sending air into the target segment. The purpose of this study was to investigate the factors associated with BJV failure.

**Methods:**

Data were retrospectively collected from 48 patients who underwent pulmonary segmentectomy with BJV between March 2014 and May 2019 at a single center. Data were compared between BJV succeeded cases and failed cases.

**Results:**

In 13 cases (27%), BJV were unsuccessful. The Brinkman index was significantly higher in failed cases (962 ± 965 failed vs. 395 ± 415 successful, *P* = 0.0067). The success rate was significantly lower when BJV was applied to the posterior basal segmental bronchus (B10) (B10: 1/5 (20%) vs others: 34/43 (79%), *P* = 0.015).

**Conclusion:**

Long-term smoking and the bronchus corresponding to the posterior basal segment might make successful performance of BJV difficult.

## Background

Recently, lung cancer surgeries have shifted from lobectomy to segmentectomy since advances in computed tomography (CT) have enabled us to detect small and early-stage lung cancers. Although segmentectomy has anatomic and functional advantages over lobectomy [1–4], the procedure is generally more technically complex than lobectomy. In particular, recognition of the segmental fissures within the pulmonary parenchyma may be difficult with unclear boundaries between adjacent segments.

For conventional segmentectomy, demarcation of the intersegmental border is generally performed by dissecting the bronchus in the diseased segment while the lung on the operation side is deflated [5]. When an anesthesiologist resumes temporarily bilateral ventilation, the lung portion other than the target segment is inflated and an intersegmental plane is developed between the segment to be removed and the segment to be preserved. A major limitation of this method is that the inflated segments become an obstacle in the narrow intrathoracic space, especially during thoracoscopic surgery.

A method that selectively inflates the resected segment using a bronchoscope connected to a jet ventilator has been introduced [6]. In contrast to the conventional method, the segment targeted for resection is inflated, which greatly reduces the degree of interference with the thoracoscopic view. The issue with this method is that the intersegmental plane can be obscure because of collateral ventilation in emphysematous lungs [2]. In addition, secretions remaining in the airway could interfere with appropriate inflation of the targeted segment. We also experienced difficulty inserting the bronchoscope to the target bronchus in some cases. Because of these problems, bronchoscopic jet ventilation (BJV) does not always successfully create a clear intersegmental plane. Several reports have shown that BJV is useful for accurate identification of the intersegmental plane during thoracoscopic pulmonary segmentectomy [6–8]. However, the intraoperative details of BJV, such as the success rate of BJV or factors associated with difficulty of the technique, have rarely been specifically mentioned in the literature. The purpose of this study was to investigate the factors related to unsuccessful identification of intersegmental plane by BJV.

## Materials and methods

This study is a retrospective observational study carried out with the approval of the institutional clinical research ethics committee of Federation of National Public Service Personnel Mutual Aid Associations, Hamanomachi Hospital (No. 2019-21). Informed consent for the procedure was obtained by surgeon from each patient. And also, we applied opt-out method to obtain consent on this study by using our hospital website.

Study subjects were consecutive patients who scheduled for thoracoscopic pulmonary segmentectomy at our hospital between March 2014 and May 2019. Sixty-nine patients were identified in this period. We reviewed the medical records of all study subjects and collected the following data: age, sex, height, weight, smoking history, pulmonary function test, preoperative CT scan, diseased pulmonary segment to be removed, operative time, intraoperative bleeding, postoperative complications, persistent air leak, and time until drainage tube removal. Persistent air leak was defined as an air leak that persisted for longer than 5 days. Brinkman index (the number of cigarettes smoked per day multiplied by the number of years of smoking) was used to estimate the cumulative dose of smoking.

Pulmonary function tests and CT scan were performed for all patients as a preoperative check-up. Obstructive ventilatory impairment was defined as forced expiratory volume in 1 s (FEV1)/forced vital capacity (FVC) less than 0.7. Patients were considered to have emphysema if emphysematous changes were observed on preoperative CT and confirmed by radiologists. Emphysematous changes were identified as hypovascular low attenuation areas (LAA) on CT. Severity of emphysema was evaluated by the Goddard classification, which is a visual scale on which LAA values are scored for each lung field: Goddard classification 1 = 0 to 25% of LAA in the lung; Goddard classification 2 = 25 to 50% of LAA in the lung; Goddard classification 3 = 50 to 75% of LAA in the lung; and Goddard classification 4 = 75 to 100% of LAA in the lung [9].

The procedure of creating the intersegmental plane by BJV was described previously [6, 7]. Briefly, an anesthesiologist inserted a 3.2- or 3.5-mm bronchoscope connected to a jet ventilator (VS 150s Jet Ventilator, Acutronic Medical System AG, Hirzel, Switzerland) into a double-lumen endotracheal tube (Shiley Broncho-Cath, Covidien, Dublin, Ireland). After the tip of the bronchoscope was positioned in the orifice of the targeted segmental bronchus, an anesthesiologist started jet ventilation. We delivered jet ventilation to patients with the following parameters: respiratory frequency 20 cycles per minute; inspiratory time 20%; FiO2 1.0. The inflation pressure was started from 0 bar and gradually increased while observing the bulging of the lung. When the delivered air filled the targeted segment, the surgeon tied the target bronchus to maintain the expansion of the segment and excised the targeted segment along the created inflation-deflation line. Ten anesthesiologists performed BJV at random.

Operative records were thoroughly reviewed to investigate whether or not the inflation-deflation line was demarcated sufficiently clearly to identify the targeted lung segment with BJV. The BJV method was considered to have been unsuccessful if there was a description in the operative records that BJV was not able to clearly outline the inflation-deflation line and alternative methods were used to pursue the surgery. In cases where BJV was unsuccessful, information about the alternative methods was obtained from the operative records.

We measured the success rate of the BJV method and analyzed the relationships between patients’ baseline characteristics and the success rate of BJV. The unpaired t test, Chi-squared test, and Fisher’s exact test were used for statistical analyses. All analyses were performed using the STATA 14.2 software (StataCorp LLC, TX USA), and P values < 0.05 were considered significant.

## Results

**Fig. 1:**
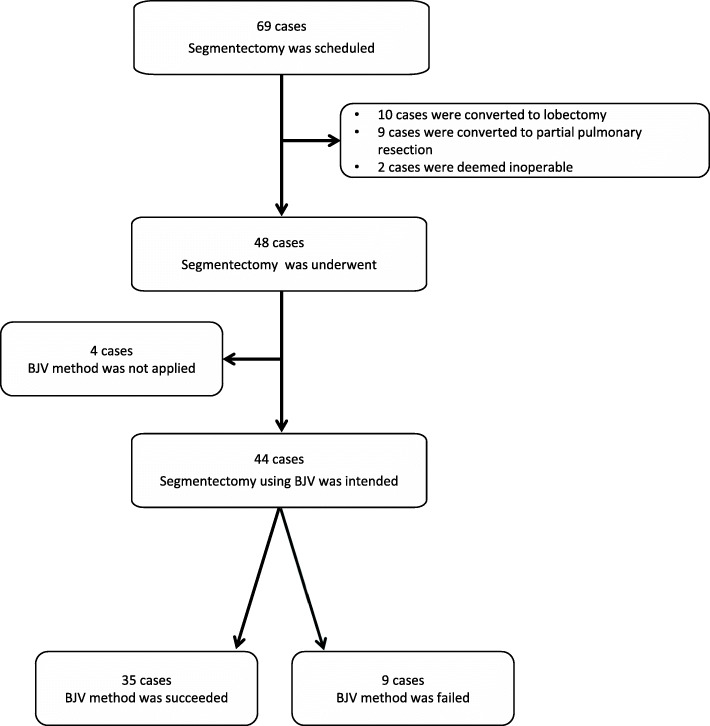
Flow chart showing patients scheduled for pulmonary segmentectomy

Segmentectomy was scheduled in 69 patients according to the responsible surgeons’ judgment regarding resectability and adequate surgical margins using a segmental approach. Of these cases, ten were converted to lobectomy during surgery because the surgical margins were not sufficient in nine cases and intraoperative lymph node biopsy was positive in one case. A further nine cases were converted to partial pulmonary resection during surgery due to insufficient surgical margins. Two cases were deemed inoperable due to severe adhesion in one and pleural dissemination in the other. Ultimately, thoracoscopic pulmonary segmentectomy was performed in 48 cases (Fig. 1).

The characteristics of the 48 patients who underwent lung segmentectomy are listed in Table 1. There were 23 women and 25 men with a median age of 68 (range 54-86) years. The median body mass index was 23 (range 17-34). Nine patients (9/48, 19%) had obstructive ventilatory impairment. Fourteen patients (14/48, 29%) had CT-diagnosed emphysema, two of whom were classified as severe emphysema by Goddard’s classification (Goddard’s classification: 3). Sixty-one percent of patients were former or current smokers, and the median Brinkman index was 380 (range 0-2500). The median total operative time was 310 min (range 207-754 min), and median bleeding during the operation was 20 mL (range 3-300 mL). The median chest tube duration was 2 days (range 1-11 days). Postoperative complications occurred in 11 patients, the most common being persistent air leak (n = 6). Other postoperative complications were pneumonia, pleural effusion, cardiomyopathy, subcutaneous emphysema, and chylothorax (Table 2).

**Table 1:** Patient characteristics

Variables	Total cases	Succeeded cases	Failed cases	
	*n* = 48	*n* = 35	*n* = 13	*P*^b^
Male	25 (52%)	19 (54%)	6 (46%)	0.748
Age (years)	68 ± 7.6	69 ± 7.8	65 ± 6.1	0.0741
BMI	23 ± 3.1	23 ± 2.6	23 ± 4.0	0.5569
Obstructive ventilatory impairment				
FEV1% < 70	9 (19%)	5 (14%)	4 (31%)	0.228
Emphysematous change in CT	14 (29%)	10 (29%)	4 (31%)	1
Smoking^a^				
Ever/current smoker	29 (61%)	21 (62%)	8 (62%)	0.787
Brinkman index	552 ± 658	395 ± 415	962 ± 965	0.0067
Brinkman index > 1000	8 (17%)	2 (6%)	6 (46%)	0.003
Operative time (min)	327 ± 99	327 ± 110	326 ± 66	0.964
Intraoperative bleeding (mL)	50 ± 70	44 ± 63	66 ± 86	0.3292
Time until drainage tube removal (day)	3.5 ± 2.4	3.5 ± 2.5	3.4 ± 1.9	0.8688
Postoperative complications (%)	11 (23%)	9 (26%)	2 (15%)	0.702
Persistent air leak (%)	6 (13%)	5 (14%)	1 (8%)	1
Targeted bronchus				
B1+2	14 (29%)	10 (29%)	4 (31%)	1
B10	5 (10%)	1 (3%)	4 (31%)	0.015

*BMI* body mass index, *FEV1%* forced expiratory volume in 1 s/forced vital capacity, *Brinkman index* the number of cigarettes smoked per day multiplied by the number of years of smoking

Continuous variables were presented as mean ± standard deviation

^a^Information about smoking was missing in one patient

^b^Student’s t test for mean difference, and Fisher’s exact test for percentage difference

**Table 2:** Postoperative complication after thoracoscopic pulmonary segmentectomy with bronchoscopic jet ventilation

Age	Sex	Targeted bronchus	BJV result	Brinkman index	FEV1% (%)	CT change	Drainage removal	Postoperative complication
77	F	1+2,3	Succeeded	0	85	−	POD 1	Pneumonia until POD5
75	M	1+2,3	Succeeded	200	75	−	POD 2	Pleural effusion/drainage on POD5
72	F	1+2,3	Succeeded	600	87	−	POD 2	Takotsubo cardiomyopathy on POD4
74	F	8	Succeeded	0	71	−	POD 8	Subcutaneous emphysema
67	F	9,10	Failed	0	74	−	POD 7	Chylothorax until POD2
73	F	1	Succeeded	400	76	+	POD 8	Air leaks until POD7, cylothorax until POD4
69	M	2	Succeeded	0	79	−	POD 11	Pulmonary fistula/pleurodesis on POD5 and POD7
65	M	3	Succeeded	880	70	+	POD 10	Pulmonary fistula/pleurodesis on POD8
81	M	6	Succeeded	0	84	−	POD 7	Pulmonary fistula/pleurodesis on POD5
74	M	9	Succeeded	800	70	+	POD 6	Air leaks until POD5, late onset pulmonary fistula on POD12, pleurodesis on POD13
54	F	9,10	Failed	1320	83	−	POD 6	Air leaks until POD5, late onset pulmonary fistula on POD14, pleurodesis on POD26

*BJV* bronchoscopic jet ventilation, *CT change* emphysematous change in CT, *POD* postoperative days

Identification of the segmental plane with BJV was successful in 35 patients (35/48, 73%) and unsuccessful in 13 (13/48, 27%). Among the 48 patients who underwent lung segmentectomy, BJV was not applied in four cases because surgeons, while isolating the bronchus, found it difficult to insert the bronchoscope into the targeted bronchus for anatomical reasons (2 cases: Left B1+2, bronchi were too small, 1 case: Left B1+2 and B3, adhesion formed after previous lung surgery, 1 case: Right B9, bronchial abnormality). In these cases, surgeons inserted an intravenous catheter (Supercath 5, Medikit, Tokyo, Japan) connected to a jet ventilator directly from the operative field into the targeted bronchus, and the inflation-deflation border was created by jet ventilation. Table 1 shows the patient factors grouped by BJV success and failure. Age, body mass index, prevalence of preoperative obstructive ventilatory impairment, and percentage of smokers were not different between the groups. The prevalence of CT-diagnosed emphysema was not significantly different between the groups; however, both patients who had severe emphysema (Goddard’s classification 3) belonged to the unsuccessful group. The Brinkman index was significantly higher in failed cases than in successful cases (962 ± 965 vs 395 ± 415, *P* = 0.0067). Those who had a Brinkman index over 1000 were significantly more frequent among failed cases (46% vs 6%, *P* = 0.003). Operative time, intraoperative bleeding, time until drainage tube removal, postoperative complications, and incidence of persistent air leak were not different between the groups. The success rate was significantly lower when BJV was applied to the posterior basal segmental bronchus (B10) compared to other regions (B10: 1/5 (20%) vs others: 34/43 (79%), *P* = 0.015). Table 3 shows the relationship between the location of the targeted bronchus and BJV. Segmentectomy was performed for any lobe, although upper lobe regions were the most common.

**Table 3:** Targeted bronchus and success of the bronchoscopic jet ventilation method

Targeted bronchus	Succeeded cases	Failed cases	Not attempted	Cumulative total of attempted cases	Cumulative total of failed cases	Cumulative percent of failed cases (%)
1	3	0		3	0	0
1+2	3	0	2	6	0	0
1+2,3	7	1	1	14	1	11
2	2	0		16	1	11
3	5	1		22	2	22
4,5	3	2		27	4	44
6	6	1		34	5	56
8	3	0		37	5	56
9	2	0	1	39	5	56
9,10	1	3		43	8	89
10	0	1		44	9	100
Total	35	9	4			

There were nine unsuccessful cases in which the inflation-deflation border by BJV was not clear enough to identify the target segment and four unsuccessful cases in which BJV was not applied because surgeons found it difficult to insert the bronchoscope. Alternative procedures were needed for these 13 BJV unsuccessful cases (Table 4). In four cases, the segmental plane was judged based on the blood vessel route and the demarcation line was marked by electrocautery. In three cases, extended segmentectomy combined with adjacent non-anatomic wedge resection was performed beyond the obscure demarcation line. In one case, a modified inflation-deflation method was attempted, i.e., after the involved bronchus was divided, temporary bilateral lung ventilation was conducted and the targeted segmental bronchus was tied to keep the gas inside, and thereafter the segment to be preserved was collapsed by resuming one-lung ventilation. In this case, the surgeon needed to perform non-anatomic resection beyond the demarcation line. In other cases, the inflation-deflation line was created by inserting a jet ventilation-connected indwelling needle directly into the target bronchus from the operative field and inflating the target segment.

**Table 4:** Unsuccessful cases of thoracoscopic pulmonary segmentectomies with bronchoscopic jet ventilation

Age	Sex	Targeted bronchus	BJV result	Brinkman index	FEV1% (%)	CT change	Alternative procedure	Drainage removal	Postoperative complication
74	M	1+2	No try	2420	71	−	Direct inflation into the bronchus	POD 3	
70	M	1+2,3	No try	1500	67	+	Direct inflation into the bronchus	POD 6	(Air leaks until POD5)
64	F	1+2	No try	0	80	−	Direct inflation into the bronchus	POD 2	
60	M	1+2,3	Failed	800	66	−	Direct inflation into the bronchus	POD 2	
72	F	3	Failed	0	76	−	Blood vessel route	POD 2	
67	M	4,5	Failed	2000	74	+	A modified inflation-deflation method	POD 5	(Air leaks until POD3)
68	M	4,5	Failed	1500	69	+	Blood vessel route	POD 2	
68	M	6	Failed	2500	65	+	Blood vessel route	POD 1	
56	F	9	No try	0	82	−	Direct inflation into the bronchus	POD 2	
67	F	9,10	Failed	0	74	−	Extended segmentectomy	POD 7	Cylothorax until POD2
54	F	9,10	Failed	1320	83	−	Extended segmentectomy	POD 6	Air leaks until POD5, late onset pulmonary fistula on POD14, pleurodesis on POD26
59	F	9,10	Failed	468	75	−	Blood vessel route	POD 3	
65	F	10	Failed	0	88	−	Extended segmentectomy	POD 3	

*BJV* bronchoscopic jet ventilation, *CT change* emphysematous change in CT, *POD* postoperative days

## Discussion

Thoracoscopic segmentectomy has increased in popularity in recent years, and several methods to identify the intersegmental plane have been proposed [10]. In our hospital, 48 thoracoscopic pulmonary segmentectomies with BJV were performed during a 5-year period. The success rate of BJV was 73%, with the Brinkman index and the location of the target bronchus being significantly related to the success rate.

Okada et al. [6] reported a technique involving selective segmental jet ventilation using a bronchoscope as a new method to detect the intersegmental plane. They completed thoracoscopic segmentectomy in 52 patients with a median operative time of 155 min (range 85-225) and without severe complications, and concluded that BJV provides an obvious intersegmental plane quickly and easily. They described detection of the intersegmental plane by BJV in addition to the path of the intersegmental veins, but did not include intraoperative details of BJV such as the success rate or factors associated with difficulty. In the present study, we found that thoracoscopic segmentectomy by BJV was successful in 35 out of 48 cases, for an acceptable success rate of 73%.

In patients with chronic obstructive pulmonary disease (COPD), it is supposed that the jet ventilation method is difficult because the inflation-deflation line could be obscure or misleading due to collateral ventilation [2]. In the present study, COPD was not related to the failure of BJV. The jet ventilation delivers extremely small tidal volumes that might not be sufficient to inflate the adjacent segment through the collateral respiratory structure. In addition, the surgeon can see the gradual inflation of the targeted segment and stop the jet ventilation upon complete inflation but before air flows into the collateral ventilation. The intersegmental line by BJV was obscure in two patients who had Goddard’s classification 3 severe emphysema in our study. The number of patients who had severe emphysema was relatively small. Further study is needed to investigate the relationship between severe emphysema and BJV success. Our data indicate that BJV is applicable to moderate emphysema, although the small number of patients in this study was a limitation.

Another difficulty with the jet inflation method is secretions remaining in the airway that can interfere with inflation of the targeted lung. In the present study, a higher Brinkman index was significantly related to failure of BJV. Sufficient delineation of the intersegmental plane by BJV could not be achieved in 75% of patients who had a Brinkman index over 1000. Long-term smoking increases mucus production and causes heavy airway secretions, which could be a cause of the lower success rate of BJV. Preoperative intervention to facilitate secretion drainage, such as pulmonary rehabilitation or anticholinergic inhalation with a nebulizer, could improve airway clearance and potentially improve BJV success.

In the present study, the success rate was significantly lower when BJV was applied to the posterior basal segmental bronchus (B10) compared to other regions. B10 is located in a very deep area; it might be difficult to effectively insert the bronchoscope into the deep smaller bronchi. During BJV procedure, surgeons helped an anesthesiologist lead the bronchofiberscope to the targeted bronchus through the light of the tip at the surgical field. According to the operative records, the bronchoscope was positioned in B10 appropriately; however, the inflation-deflation line was not clear enough to identify the segmental plane. It is possible that the deeper bronchus might be more vulnerable to airway secretion. In addition, the anatomy of S10 and adjacent segments such as superior (S6) and medial basal (S7) varies among patients, which might make it difficult to clearly identify the intersegmental plane of the posterior basal segment [11].

The right and left lung anatomy are asymmetrical. The BJV success rate was not different between right and left. There are three segments in the right upper lobe: apical (B1), posterior (B2), and anterior (B3). On the other hand, there are four segments in the left upper lobe: apicoposterior (B1+2), anterior (B3), inferior lingual (B4), and superior lingual (B5). Regarding difficulty reaching the target lesion with the bronchoscope, B1+2 was considered to be the most difficult for insertion because of its sharp angle [12]. During surgery, bronchoscopy may be further complicated by the patient’s lateral decubitus position. In our study, four cases including three B1+2 were not applied BJV because the surgeon found that the target bronchus was anatomically too difficult for bronchoscope insertion. The success rate of BJV was not different between B1+2 and the other regions.

Various methods have been proposed to identify intersegmental planes during thoracoscopic pulmonary segmentectomy [10, 13]. In the slip knot method, a specific ligation (slip knot) is applied to the target segment bronchus followed by bilateral ventilation, and then the knot is slipped to close the bronchus [14–16]. This method does not require a jet ventilator and anesthesiologist who manipulate bronchoscope; however, in some cases, it takes time to obtain lung collapse in the preserved segments. Other methods use indocyanine green (ICG), which is a green dye visible under regular white light and visible as fluorescence by near-infrared light. Misaki et al. reported intravenous ICG injection after clamping the pulmonary arteries perfusing the target segment [17–19]. Under fluorescence thoracoscopy, the target segment is visualized as a dark area while the lung perfused with ICG appears as a bright area. Limitations of this method are that the duration of intravascular ICG visualization is short because ICG is rapidly washed out, and that fluorescence thoracoscopy is not yet widely available. The BJV method needs a skillful anesthesiologist and a jet ventilator. A jet ventilator provides high-frequency jet ventilation (HFJV) for laryngeal surgery and management of the difficult airway which have potential hazards to cause barotrauma [20]. In the BJV method, a jet ventilator is used to send small volume of air into the targeted segment through a bronchoscope, not for respiration (gas exchange). Therefore, inspiratory driving pressure and frequency is much less than HFJV. Driving pressure setting for HFJV is 1–3 bar [20], whereas pressure needed for BJV is less than 0.6 bar. A jet ventilator enables an anesthesiologist to increase driving pressure gradually and stop inflation immediately with one hand while he/she holds a bronchoscope with the other hand. Surgeons who can monitor the lung directory from the operative field warn hyperinflation during BJV. Close collaboration between the anesthesiologist and the surgeon is essential to safely complete the procedure.

Although there were thirteen unsuccessful BJV cases, neither operative time nor intraoperative bleeding was higher in these cases compared to the successful cases. We consider this was because alternative approaches were promptly applied. Typical difficulties encountered during BJV are insertion of the bronchoscope to the targeted bronchus and inflation of the targeted segment with jet ventilation. Proper insertion can be quickly confirmed from the surgical field by the bronchoscope tip light, and surgeons can also directly see the targeted bronchus and estimate the anatomical feasibility of inserting the bronchoscope. Inflation of the targeted segment can also readily be confirmed. During jet ventilation, the surgeon can see the gradual inflation of the targeted segment and either clear visualization of the intersegmental line or failure to create the inflation-deflation line owing to collateral ventilation or airway secretion. These features of BJV help the operator to make a judgment on whether the BJV method is feasible or not, and to promptly change tactic when necessary.

Incidence of postoperative complications was not different between BJV success cases and BJV failed cases. In the present study, air leaks and pulmonary fistula were the most common issue. In BJV failed cases, adjacent non-anatomic wedge resection was performed; however, persistent air leaks did not occur except one case. Smoking, pleural adhesion, stapling length, and early postoperative drainage were reported to be a significant risk factors for persistent air leaks after pulmonary resection [21, 22]. These factors might play a dominant role on incidence of the postoperative air leaks. Direct inflation into the bronchus by puncturing with a needle has been reported to cause massive air embolism, most likely resulting from direct injection of air into an adjacent pulmonary vein [14, 16]. Although we did not have any complication with the alternative method, great care is essential as this approach. Among those who had surgery except segmentectomy (n = 21) and those who were not applied BJV (n = 4), postoperative complications were seen in 5 patients. Incidence of postoperative complications was not different between BJV cases and non-BJV cases (11/44, 25% vs 5/25, 20%, *P* = 0.636).

There were some limitations in this study. The BJV technique might depend on the skill of the anesthesiologist; however, BJV was not performed by a single anesthesiologist in the present study. Ten anesthesiologist performed BJV during the 5-years study period and there was not obvious individual difference in the BJV success rate. All anesthesiologist had more than 4 years of experience and more experienced anesthesiologist supervised them if they needed. Although bronchoscopy is the widespread practice, the BJV method requires a clear knowledge of tracheobroncial anatomy with the fiberoptic bronchoscope. A video screen monitor to allow sharing the views was used whenever possible in the present study. Enhanced bronchoscopic view and communication between anesthesiologists and surgeons helped us succeeded BJV technique.

Another limitation is the small number of the cases. Although we included all patients scheduled for pulmonary segmentectomy during 5 years, there were only two patients with severe CT-diagnosed emphysema. Confounding may also exist in the present study because data were gathered retrospectively. Statistical adjustments were not performed due to the small sample size. The observed relationships between difficulty of BJV and long-term smoking or deep bronchus seem clinically reasonable. Further research including larger numbers of patients is needed.

## Conclusions

In conclusion, our data demonstrate that BJV is a sound option for identifying the intersegmental plane during thoracoscopic segmentectomy and can be applied to patients with moderate emphysema. Predictors of BJV failure are long-term smoking and posterior basal segment. The BVJ method seems to be a feasible and reasonable procedure for thoracoscopic segmentectomy.

## Data Availability

The data that support the findings of this study are available from the corresponding author on reasonable request.

## Abbreviations

BJV: Bronchoscopic jet ventilation
CT: Computed tomography
FEV1: Forced expiratory volume in 1 s
FVC: Forced vital capacity
LAA: Low attenuation areas
HFJV: High frequency jet ventilation
